# A Long Cord

**Published:** 1885-09

**Authors:** R. G. Williams

**Affiliations:** Whitney, Texas


### A LONG CORD.

*By R. G. Williams, M. D., Whitny, Texas.*

For Daniel's Texas Medical Journal.

MRS. D. J. T. was delivered, August 7th, 1885, of a healthy female child, after an unusually easy and short labor. Afterbirth immediately followed expulsion of child. The cord was thrice around the child's neck, and tied in a knot midway its length. This cord, seemingly of great length, was carefully measured, and found to be from its severed end to its insertion fifty-eight inches. Two inches was left on child, giving sixty inches as entire length.

STATE MEDICAL EXAMINER K. OF H.: Dr. Jas. D. Osborn, of Cleburne, Texas, was reelected State Medical Examiner K. of H., by a large majority, at the recent meeting of the Supreme Lodge of Knights of Honor. Dr. Osborn, who is also chairman of State Finance Committee of the International Medical Congress, informs us he will soon send us the names of the committee, for publication, as soon as he completes his list of appointments—a member for each of the 226 counties.

